# Investigating the isolated effects of a soccer-specific mental fatigue manipulation on different task types

**DOI:** 10.3389/fpsyg.2025.1655221

**Published:** 2025-09-18

**Authors:** Helena Weiler, Fabienne Ennigkeit, Stefan Altmann, Lena Steindorf, Jan Spielmann, Chris Englert

**Affiliations:** ^1^Department of Sport Psychology, Goethe-Universität Frankfurt am Main, Frankfurt, Germany; ^2^Department for Performance Analysis, Karlsruhe Institute of Technology, Karlsruhe, Germany; ^3^Department of Physiology, TSG ResearchLab gGmbH, Zuzenhausen, Germany

**Keywords:** mental fatigue, mental effort, technical performance, cognitive performance, soccer, transfer effects, team sport

## Abstract

**Introduction:**

Mental fatigue negatively impacts athletic performance, but commonly employed tasks like the Stroop task often lack ecological validity. This study aimed to validate a modified, soccer-specific Footbonaut task as a mental fatigue manipulation and examine its effects on following tasks representing task-specific, near-, and far-transfer domains.

**Methods:**

A randomized, counterbalanced within-subject design was implemented with *n* = 24 soccer players. Participants completed a Footbonaut task (task-specific), the LSPT (near-transfer), and the Stroop task (far-transfer) before and after mental fatigue manipulation via the Footbonaut.

**Results:**

Inconsistencies emerged between interaction effects and post-hoc tests, showing no clear negative effect of the manipulation on accuracy or response times. The employed mental fatigue manipulation did not differentially affect the three tasks, indicating a lack of transfer effects.

**Discussion:**

Although mental fatigue was not successfully induced by the sport-specific Footbonaut task, the findings emphasize the need for ecologically valid, innovative methods to better understand mental fatigue in sports.

## 1 Introduction

According to the psychobiological model, mental fatigue (MF) is conceptualized as a psychobiological state which is often experienced during, or after, working on mentally effortful tasks over extended periods of time (Marcora et al., 2009) — such as maintaining focus over prolonged periods or continuously making decisions while considering complex team tactics. MF can exert its effects on a subjective (e.g., feelings of tiredness, lack of energy; Van Cutsem et al., 2017), physiological [e.g., changes in heart rate (HR); e.g., Marcora, 2019] and behavioral level (e.g., reduced accuracy and/or response time; Smith et al., 2017). There is considerable evidence indicating that MF impairs different types of performance, particularly in sports characterized by high mental and cognitive demands, such as soccer (Li et al., 2025; Thompson C. J. et al., 2020), golf (Wang et al., 2021), or basketball (Cao et al., 2022).

In soccer, fatigue has been primarily studied from a neuromuscular and metabolic perspective (i.e., physical fatigue), while only limited attention has been given to mental and cognitive factors (Kunrath et al., 2020a). However, soccer is considered to place high mental demands on players, such as sustained attention, response inhibition, rapid decision-making, tactical planning and dealing with pressure. Soccer players are required to remain vigilant, while distracting but situationally irrelevant information needs to be ignored (e.g., the crowd) and tactical behavior has to be adjusted in an ever-changing environment over the course of a match or during practice (Kunrath et al., 2020a, 2020b). Additionally, players have to make fast and accurate decisions in high-pressure situations (Bian et al., 2022). In order to deal with these multiple mental demands, players must invest mental effort which consequently can trigger perceptions of MF (Kunrath et al., 2020b; Smith et al., 2018).

A frequently used research approach to investigate the effects of MF on soccer-specific performance parameters is the sequential two-task paradigm (Englert and Bertrams, 2014). First, participants work on a mentally demanding task which is utilized to induce MF (i.e., independent variable). Subsequently, a second mentally demanding task is performed to assess the effects of MF on a subjective, physiological and behavioral level (i.e., dependent variable). Previous studies revealed detrimental effects of MF on several soccer-specific performance indicators, for instance on physical (e.g., reduced intermittent running performance, Smith et al., 2015), cognitive (e.g., decision-making; Trecroci et al., 2020), technical (e.g., defensive/offensive techniques; Sun et al., 2022), and tactical performance (e.g., less defensive coverage; Kunrath et al., 2020b).

While sport-specific tasks have been frequently used as dependent variables in MF research in the past, researchers often adopt sport-unspecific, computerized cognitive tasks to induce MF (Coutinho et al., 2017; Kunrath et al., 2020a). One of the most frequently applied MF tasks in sport and exercise research is the (modified) Stroop task (see also Van Cutsem et al., 2019), which involves inhibitory control and decision-making processes both of which are also relevant in several types of sport (Smith et al., 2018). One of the major advantages of these highly standardized, laboratory-based manipulation tasks is that they allow controlling for various confounding variables (e.g., muscle fatigue; Habay et al., 2021) and isolating the effects of MF on performance. Despite its effectiveness in inducing MF, laboratory-based manipulation tasks have recently been challenged for their lack of ecological validity (Bian et al., 2022; Coutinho et al., 2017; Habay et al., 2021; Weiler et al., 2025b). The extent to which these rather artificial tasks correspond to the real-world mental and cognitive demands that athletes face during training and competition has been questioned. To bridge this gap between research and the applied field, several researchers and practitioners have called for the development of ecologically valid and sport-specific tasks to further demonstrate the relevance of MF in sports (Smith et al., 2018; Thompson C. et al., 2020; Trecroci et al., 2020; Weiler et al., 2025b). Furthermore, increasing the sport-specificity of MF manipulation tasks facilitates a clearer differentiation between MF and related constructs such as boredom (Wolff et al., 2022). Previous research suggests that, in athletes, prolonged engagement in computerized, sport-unrelated tasks can increase perceptions of boredom over time, while more ecologically valid, sport-specific tasks foster greater intrinsic motivation and engagement, thereby (potentially) reducing the likelihood of boredom-related confounds (Martarelli et al., 2023; Milyavskaya et al., 2021).

In soccer research in particular, there have been several attempts to address the low levels of ecological validity in MF research by developing more sport-specific study designs. The central aim is to investigate the extent to which the sport’s mental and cognitive demands contribute to increased perceptions of MF in training, competition or camp settings (Thompson et al., 2019). The results of recent qualitative interviews shed light on both these sport-specific (e.g., continuous decision-making during a game) and sport-unspecific (e.g., media engagement) triggers of MF (Russell et al., 2019; Weiler et al., 2025a).

Using a quantitative-experimental approach, Coutinho et al. (2017) induced MF in soccer players by having them perform a 20-min motor coordination task. The players were asked to perform various types of ladder drills (e.g., jumping, running backwards) while juggling a tennis ball to increase the mental demands of the task. Following completion of this task, players reported higher levels of MF and showed physical and tactical performance decrements during subsequent soccer small-sided games. Ciocca et al. (2022, see also Greco et al., 2017) implemented commonly used pre-match routines (e.g., film-based tactical sessions, smartphone use) to induce MF in soccer players, which led to higher perceptions of MF and impaired subsequent motor performance.

Expanding on these approaches, Bian et al. (2022) and Weiler et al. (2025b) developed novel MF manipulation tasks that induce MF during football play itself, going beyond routines performed before training or matches (e.g., coordination drills or tactical sessions). This marks an important shift toward studying how MF arises from the actual demands of football, rather than from isolated tasks completed beforehand. Bian et al. (2022) used the Loughborough Soccer Passing Test (LSPT), a multifaceted soccer-specific skill test, to induce MF in soccer players. In this test, players are instructed to receive and pass 16 balls in a randomized passing order under restricted time and space conditions. Thus, the task contains both, soccer-specific (e.g., passing, dribbling, control) and mental demands (e.g., decision-making, sticking to rules) that soccer players are commonly confronted with during training and competition (González-Víllora et al., 2022; Kunrath et al., 2020a). Another promising approach was developed by Weiler et al. (2025b). They utilized the Footbonaut, a skill assessment and training tool, to induce MF under ecologically valid conditions. The Footbonaut consists of an artificial turf pitch surrounded by ball machines and illuminated target fields. In the regular version, players have to receive and then pass a ball to an illuminated target. However, in the modified, mentally demanding version of the standard Footbonaut task, players no longer responded to a single predetermined target. Instead, they had to select one of three potential target fields based on frequently changing rules. This manipulation increased the cognitive demands of the task by requiring players to continuously update their decisions and inhibit automatic responses—both of which are considered key triggers of MF (e.g., decision-making, inhibition). The studies of Bian et al. (2022) and Weiler et al. (2025b) provide initial evidence of how MF can be experimentally manipulated in a more soccer-specific manner. Both novel manipulation tasks led to impaired performance in subsequent soccer-specific tasks (e.g., LSPT).

Interestingly, in both studies there were no statistically significant effects of MF on general cognitive performance (e.g., Stroop task). Weiler et al. (2025b) draw on the framework of perceptual-cognitive training to explain these results. According to this framework, training a specific perceptual-cognitive skill (e.g., decision-making) influences subsequent performance at three levels: (1) task-specific transfer, referring to improvements in the identical task; (2) near-transfer, referring to improvement in similar tasks, which require the same perceptual-cognitive skills; (3) far-transfer, referring to improvement in unrelated tasks, which require the same perceptual-cognitive skills (Zentgraf et al., 2017). Previous research provides strong evidence for the occurrence of performance improvements in the identical task (task-specific) or in similar tasks (near-transfer). However, the effects of perceptual-cognitive training on unrelated tasks (e.g., sport-specific performance; far-transfer) remain inconclusive (e.g., Fransen, 2022; Gobet and Sala, 2023). Drawing on these theoretical considerations and empirical findings, it is conceivable that MF effects induced by sport-specific manipulations (e.g., repeated interval LSPT; Bian et al., 2022; mentally demanding task in the Footbonaut; Weiler et al., 2025b) primarily manifest at the task-specific (i.e., LSPT performance; Bian et al., 2022) or the near-transfer level (i.e., LSPT performance; Weiler et al., 2025b), while having little to no impact on performance in unrelated tasks (i.e., far-transfer Stroop performance; Bian et al., 2022; Weiler et al., 2025b).

The present study aims to replicate and extend the previous findings on the validity of the aforementioned mentally demanding Footbonaut task developed by Weiler et al. (2025b). For that cause, the effects of this soccer-specific manipulation task on performance (i.e., accuracy, response time) in three different types of tasks – the Footbonaut (identical task; task-specific), the LSPT (soccer-specific; near-transfer) and the Stroop task (general cognitive; far-transfer) – were tested. It is hypothesized that performance (accuracy and response/processing time) in each task will be impaired after having worked on the mentally demanding version of the Footbonaut task: lower passing performance and decreased processing speed in the Footbonaut, increased penalty time and increased movement time in the LSPT, and more errors and slower response latencies in the Stroop task. Due to the mixed findings on far-transfer effects in previous studies, it will further be tested whether the three different types of tasks are influenced to different degrees by the MF induced by the Footbonaut task.

## 2 Methods

### 2.1 Participants

The results of an *a priori* power analysis (G∗Power software version 3.1.9.7; Heinrich-Heine-Universitat Düsseldorf, Germany; Faul et al., 2009) revealed that a sample of 24 soccer players was required to detect a moderate effect of MF (Brown et al., 2019; analysis: within-subjects analysis of variance; parameters: *f* = 0.30, *α* = 0.05, 1 − *β* = 0.80, *r*_repeated_ measures = 0.50, *ε* = 1). To account for potential dropouts, a total of 28 adult male soccer players were recruited. Four players had to be excluded from the testing period due to illness and injuries. The final sample consisted of 24 well-trained, competitive soccer players (age: *M =* 24.3, *SD* = 4.2) classified as tier athletes (McKay et al., 2022) from the fifth (*n =* 3) and sixth (*n =* 21) highest German soccer leagues, including 5 forwards, 10 midfielders, and 9 defenders. Goalkeepers were excluded, due to the distinct position-specific demands (Sarmento et al., 2024). Following an initial health screening, it was ensured that all participating players were free of (chronic) diseases, injuries or color vision deficiencies. Written informed consent from each player was obtained before commencing the study. Participation in the study was voluntary and could be withdrawn at any time of the experimental procedure without providing any reason. The study was approved by the local ethics committee (November 2024, Approval number 2024–53) and carried out in accordance with the Helsinki declaration.

### 2.2 Study design and procedure

**Figure 1:**
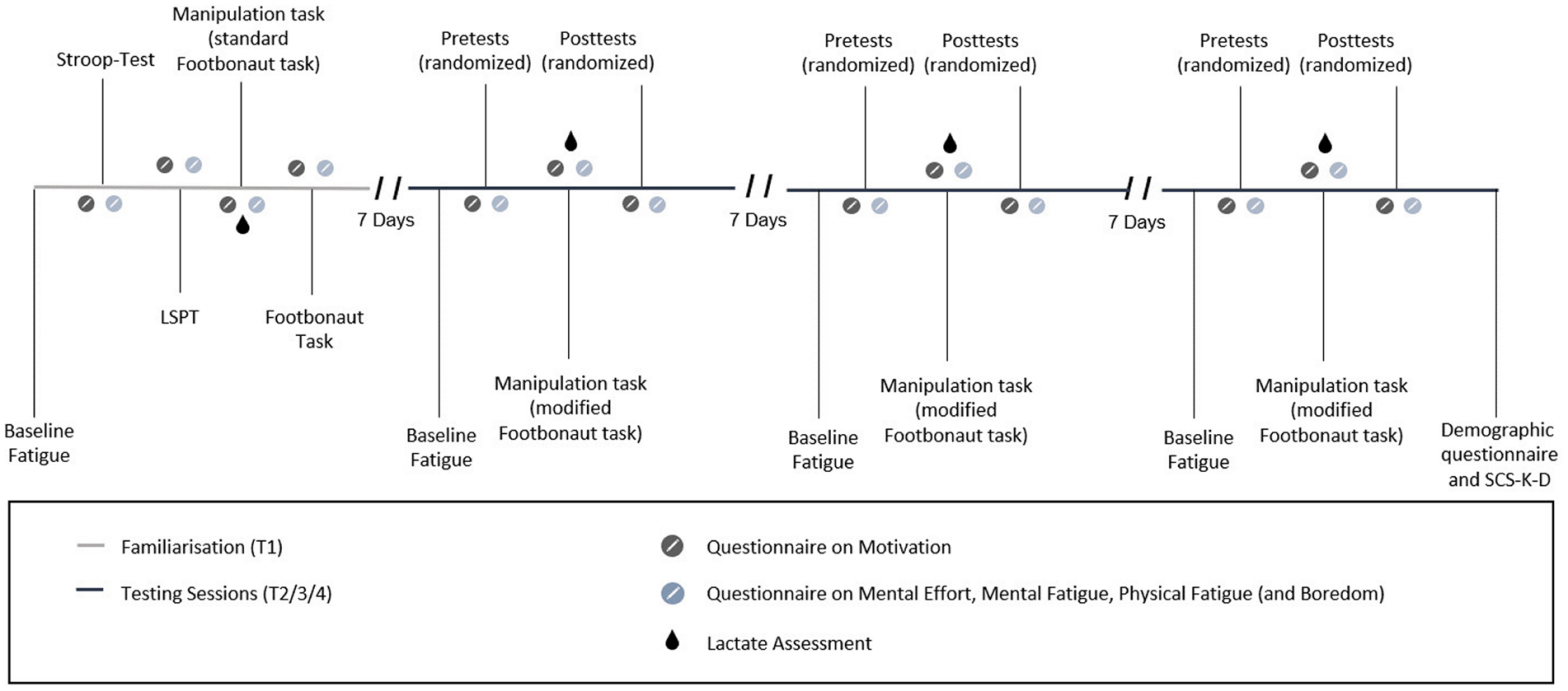
Timeline of testing sessions (T2-T4).

The preregistered study^1^ had a randomized, counterbalanced, within-subject experimental design with four times of measurement (T1–T4) with each visit being separated by a 7-day interval (see Figure 1 for a graphical representation of the entire procedure). Each time of measurement took place at the same time of the day in order to minimize the potential influence of circadian rhythms. While the procedure for the first time of measurement was the same for each player and was used for familiarization purposes, T2, T3 and T4 included the three different experimental conditions which were performed in a randomized and counterbalanced order.^2^

During familiarization, players were provided with standardized definitions of mental and physical fatigue to ensure a common understanding of both concepts (see also Weiler et al., 2025a), and exposed to all testing and assessment procedures, to minimize potential learning effects (for this procedure, see Bian et al., 2022; Weiler et al., 2025b). Additionally, players performed a 30-min standard task in the Footbonaut (Beavan et al., 2018; Saal et al., 2018) to obtain baseline values for physical fatigue (for more information, see Control Measures).

At T2, T3 and T4, players were asked to work on: (1) a questionnaire on their health status (Thomas et al., 1992); (2) self-reports to assess baseline mental and physical fatigue [via Visual Analogue Scales (VAS); Thompson C. et al., 2020]; (3) a pretest on one of the three dependent variables (for more information, see Dependent Variables); (4) a mentally demanding task in the Footbonaut to experimentally manipulate MF (for more information, see Mental Fatigue Manipulation); and (5) a posttest (consisting of the same task which was performed in the pretest). Further self-reports were collected prior to (motivation) and after (mental effort, mental fatigue, physical fatigue) the pretest, the manipulation task and the posttest. Additionally, boredom was assessed after the manipulation task.

A modified version of the standard Footbonaut task was used, with the manipulation task kept identical across all measurement time points (Weiler et al., 2025b). The pre- and posttests varied across the three times of measurement in a randomized order and represented the three experimental conditions (Footbonaut, LSPT, and Stroop task; for more information, see Dependent Variables). They were applied to separately assess task-specific performance (Footbonaut; identical task), soccer-specific performance (LSPT; near-transfer), and general cognitive performance (Stroop task; far-transfer), enabling an isolated examination of the potential effects of MF on the individual performance in these tasks. At the last time of measurement, players completed a brief demographic questionnaire (e.g., age, soccer position), along with a trait questionnaire to measure their level of trait self-control (SCS-K-D; Bertrams and Dickhäuser, 2009). As the latter is not relevant to this study, it will not be discussed in further detail. After the experiment was completed, participants were debriefed, given the opportunity to ask questions, and thanked for their participation.

### 2.3 Control measures

The German version of the Physical Activity Readiness Questionnaire (PARQ; Thomas et al., 1992), consisting of seven dichotomous items (e.g., “Do you have a bone or joint problem that could be worsened by a change in your physical activity?,” yes vs. no), was used to assess players’ health status. As no player reported the onset of any of the physical symptoms, no participant had to be excluded from the study.

VAS, 100 mm horizontal line scales, ranging from 0 (“not at all”) to 100 (“maximum”), were employed to measure the participants’ current perception regarding the following five parameters (for this approach, see also Van Cutsem and Marcora, 2021; Thompson C. et al., 2020): (1) motivation (“Please indicate how motivated you are regarding the upcoming task”), (2) mental fatigue (“Please indicate the level of your current state of mental fatigue”), (3) physical fatigue (“Please indicate the level of your current state of physical fatigue”), (4) mental effort (“Please indicate your current state of perceived mental effort”), and (5) boredom (“Please indicate how bored you currently feel”).

The HR and blood lactate concentration (BLa) were measured as indicators for physical fatigue (Djaoui et al., 2017; Miguel et al., 2021). HR was monitored using an HR chest strap (Polar H10 Sensor, Kempele, Finland). To measure BLa, capillary blood samples were collected from the hyperemic earlobe (Biosen C-Line Sport, EKF-diagnostic GmbH, Barleben, Germany). HR and BLa were assessed five times during the manipulation task in the Footbonaut (T2–T4), once at each time of measurement: first, immediately before performing the manipulation task and then after each set of 80 balls played. The same procedure was applied during the familiarization phase (T1), where players performed a 30-min standard Footbonaut task consisting of four sets of 80 balls, with HR and BLa recorded before and after each set (see also Weiler et al., 2025b). This procedure aimed to investigate if the mentally demanding manipulation task used in the current study was comparable in terms of the physical load with the 30-min standard task in the Footbonaut to control for potential unintended effects of physical effort.

### 2.4 Mental fatigue manipulation

The MF was experimentally manipulated by the previously described soccer-specific passing task in the Footbonaut. For this purpose, a modified version of the standard task in the Footbonaut (derived from Weiler et al., 2025b) was used.

**Figure 2:**
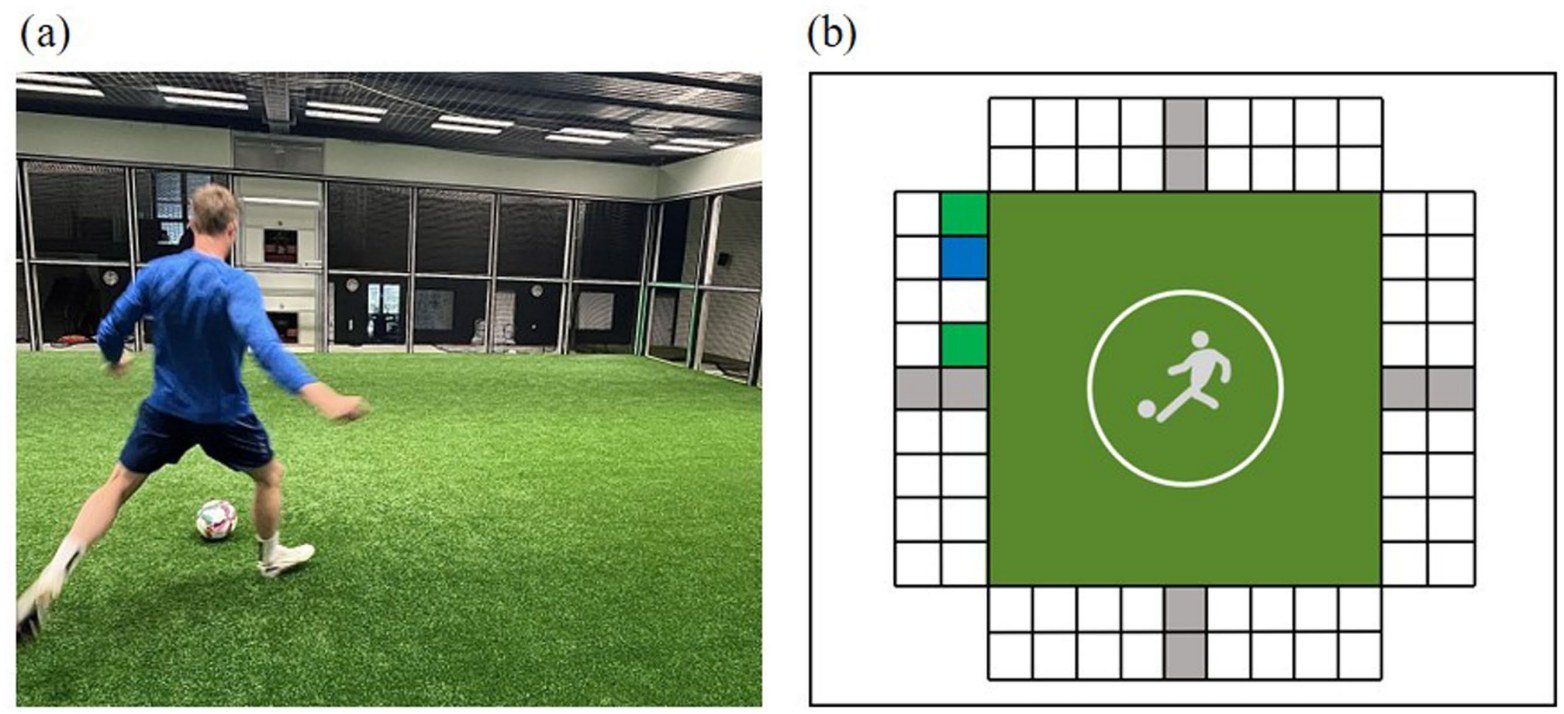
**(a)** Photograph of the Footbonaut in use, showing a player interacting with the system during a training session. **(b)** Graphical representation of the modified task in the Footbonaut.

The Footbonaut comprises an artificial turf pitch surrounded by four grid walls (C-Goal GmbH, Berlin, Germany). Each wall consists of 18 targets (9 upper, 9 lower) and two ball machines (one upper, one lower), which can be visually activated by light-emitting diodes. A graphical representation of the setup is shown in Figure 2. For execution of the standard task, the players position themselves in the center of the turf pitch while the experimenter starts the task. The direction of the incoming ball is indicated by an audio-visual signal emitted by the ball machine. The ball is played with a delay of 800 ms while another audio-visual signal activates the respective target field at the same time. The player’s task is to receive the ball and pass it through the corresponding target field. Once the ball has passed a field’s light barrier (regardless of whether it was the target field or not), another audio-visual signal follows from the ball machine, indicating the direction of the next ball (for more information on the setup and the standard procedure, see Beavan et al., 2018; Weiler et al., 2025b).

**Figure 3:**
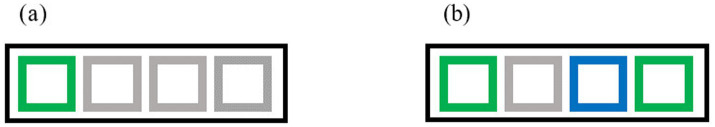
Detailed graphical representation of the **(a)** standard and **(b)** modified task in the Footbonaut.

To induce MF (T2, T3, and T4), a modified version of the standard task in the Footbonaut was used in the current study. The manipulation task lasted approximately 30 min and included 320 trials, divided into four sets of 80 balls each. The 30-min duration was chosen based on van Cutsem et al.’s (2019) conclusion that tasks should last at least 30 min in order to reliably induce MF. The task included additional decision-making and inhibition processes in order to increase the mental demands of the standard task in a soccer-specific way. During the task, only the lower fields and the lower ball machines were activated and the targets were only announced by a visual signal. Instead of one target field, a total of three potential target fields were activated per trial (see Figure 3 for a graphical representation). Two of the fields were illuminated in the same color and one field in a different color. The instruction regarding the relevant target field varied across the four sets in the Footbonaut in order to maintain the mental demands, as well as to prevent reduced motivation or perceptions of boredom (Milyavskaya et al., 2021; for the exact instructions, see Supplementary Data Sheet 1). A 30-s break was taken between sets to assess the physiological parameters HR and BLa.

### 2.5 Dependent variables

The dependent variables were recorded as a part of the pre- and posttests. Each task lasted 3 min.

#### 2.5.1 Stroop task

To assess the effects of MF on general cognitive performance (i.e., far-transfer effect), a three-minute version of the Stroop task, programmed in OpenSesame (version 4.0.5.), was performed on a regular computer screen (see also Bian et al., 2022; Smith et al., 2016). In the current study, a series of colored words (red, blue, yellow, and green) in different font colors (red, blue, yellow, and green) was presented randomly on a black background (100% incongruent). The players were required to respond to each word’s font color rather than its semantic meaning as accurately and quickly as possible by pressing the corresponding key on the keyboard (e.g., if the word yellow appears in blue, the button “blue” is to be pressed). However, in order to increase the difficulty of the task, an exception was made: In case the word appeared in a red font color, the players had to respond to the semantic meaning (e.g., if the word red appears in blue, the button “red” is to be pressed; Pageaux et al., 2015). Once a response was provided, the stimulus disappeared from the screen, followed by the presentation of a new stimulus. The task aborted automatically after 3 min. Players’ cognitive performance was evaluated based on their absolute number of errors (accuracy) and average response latencies (time).

#### 2.5.2 Loughborough soccer passing test (LSPT)

**Figure 4:**
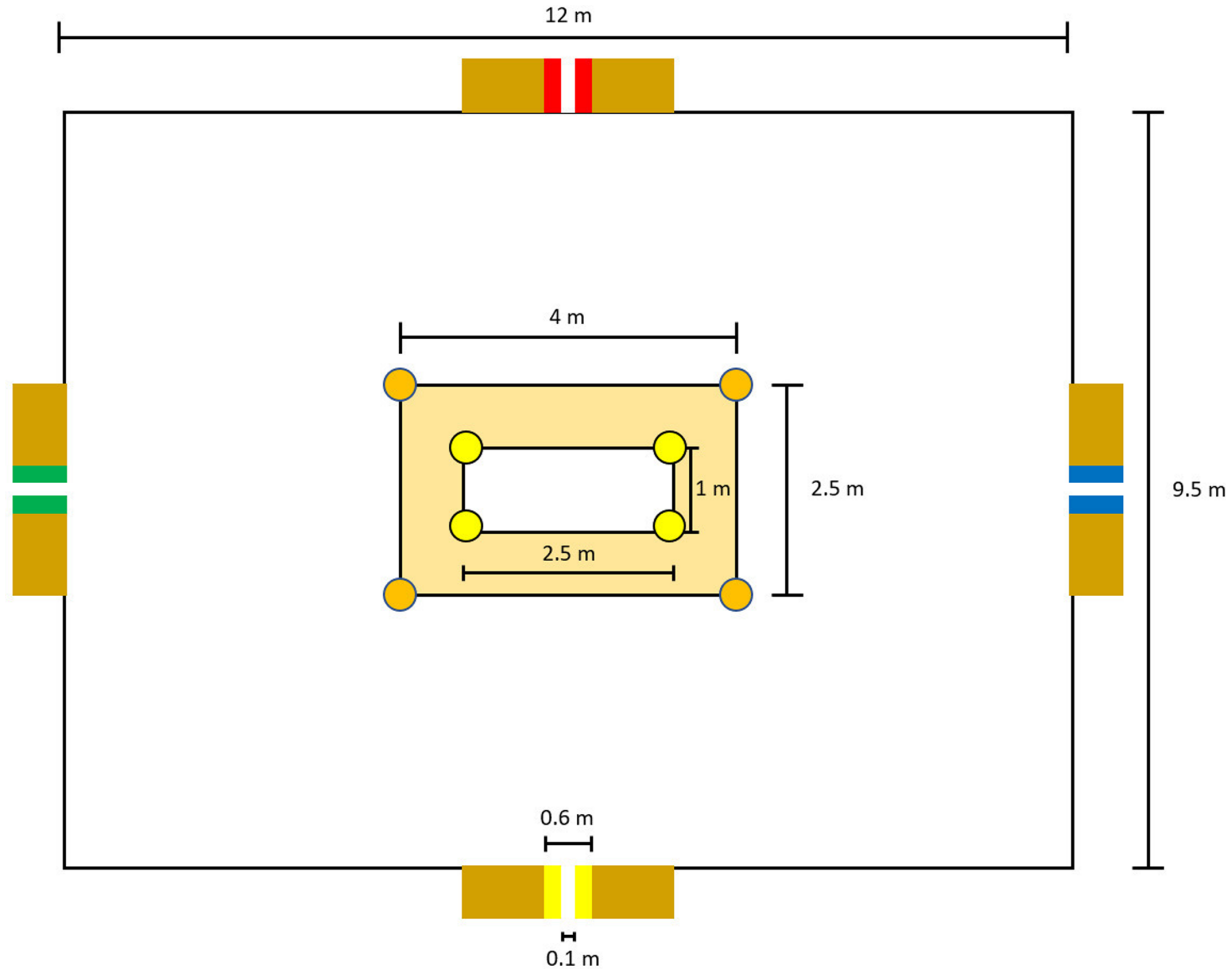
Graphical representation of the LSPT.

The LSPT was used to measure player’s soccer-specific performance (i.e., near-transfer effect; see also Bian et al., 2022; Weiler et al., 2025b). The setup of the LSPT consists of the playing zone (12 × 9.5 m), including the central zone (2.5 × 1 m) and the passing zone (4 × 2.5 m), which is surrounded by four colored passing targets (a green, blue, yellow and red standard bench; see Figure 4). Players started in the center of the rectangular playing zone and responded to the experimenter’s audible signal (e.g., “Blue”) which indicated the target/passing direction. One set of the LSPT consisted of 16 passes, using a randomized order (see Footnote 2) of the aimed passing targets. To assess soccer-specific performance, three sets of the LSPT were conducted, with a standardized 75-s break between each set. The players were asked to perform each set as quickly and accurately as possible. Using video recordings, the performance of the players was subsequently evaluated by an independent rater. While the movement time (in seconds) was the total time required to complete the task, the penalty time (in seconds) was dependent on an accurate completion of the task. Penalty time was added for errors (passing inaccuracy, e.g., passes outside the passing area) and deducted for highly accurate passes (hitting the white stripes positioned in the middle of the colored targets) (for more information, see Supplementary Material ESM 1). Players’ soccer-specific performance in the LSPT was evaluated based on their penalty time (accuracy) and movement time (time). For the statistical analyses, total scores were computed by summing the individual scores from the three trials for each performance parameter.

#### 2.5.3 Footbonaut task

A three-minute version of the modified Footbonaut task was used to assess task-specific performance (derived from Weiler et al., 2025b). This version of the Footbonaut task was identical with the Footbonaut task used for the manipulation and differed only in the duration of execution and in terms of the instruction. Players passed 40 balls and the instructions regarding the target field (play the green light that is further from the blue light) were kept constant. The players were instructed to perform the task as quickly and accurately as possible. Players’ soccer-specific performance in the Footbonaut task was evaluated based on their average passing performance (accuracy) and average processing speed (time).

### 2.6 Statistical analyses

All statistical analyses were conducted using JASP, version 0.18.3 (Amsterdam, Netherlands). The significance level was set at *p* = 0.05. Effect sizes were interpreted according to Cohen (1988). First, the data was examined for potential order and habituation effects. Afterwards, we checked whether the subjective control measures changed from pre- to posttest in all three conditions. Then, we compared the change in HR and BLa over the course of the mentally demanding Footbonaut task in the three conditions with the change in HR and BLa in the mentally less demanding Footbonaut task during the familiarization to control for unintended physiological effects. Finally, we tested the main hypotheses.

To enhance clarity, the statistical hypothesis tests used are presented directly within the results section. When two-way repeated measures ANOVAs are reported, the focus will be on the interaction effects relevant for testing the hypotheses. Details on the corresponding main effects and descriptive statistics can be found in ESM 3: Supplementary Tables S4–S8. We tested the assumption of sphericity using Mauchly’s test and applied the Greenhouse–Geisser correction whenever this assumption was violated. When significant interactions emerged, we performed post-hoc tests with Tukey correction.

## 3 Results

### 3.1 Preliminary analyses

To analyze the data for potential order effects,^3^ we conducted two separate two-way repeated-measures ANOVAs [Time of measurement (T2 vs. T3 vs. T4) × Time (pre vs. post)], one for accuracy and one for response time parameters. Prior to analysis, the outcome variables were z-standardized (for more information, see Main analyses). It was investigated whether players’ test performance differed significantly across the different times of measurement. We did not observe any order effects on the (performance-related) dependent variables (see ESM 2: Supplementary Tables S1–S3).

Furthermore, we analyzed the data for potential habituation effects. Specifically, we examined whether the manipulation task in the Footbonaut was perceived as less mentally demanding, less mentally and physically fatiguing, or more boring across the times of measurement. For this purpose, a repeated-measures ANOVA [Time of measurement (T2 vs. T3 vs. T4) × Subjective Measure (mental effort vs. mental fatigue vs. physical fatigue vs. boredom)] was conducted. No habituation effects were found in the subjective measures regarding the manipulation task in the Footbonaut (see ESM 2: Supplementary Table S1).

### 3.2 Control measures

Comprehensive descriptive statistics and further details concerning the main effects for the subsequent analyses of the control measures are provided in ESM 3: Supplementary Tables S4–S6.

#### 3.2.1 Subjective measures

Based on findings of previous research demonstrating that tasks with high mental demands elicit perceptions of MF (e.g., Van Cutsem and Marcora, 2021; Habay et al., 2021), we assumed that the experimental manipulation leads to increased perceptions of mental effort, mental fatigue and physical fatigue and decreased perceptions of motivation regarding the subsequent performance test (compared to the corresponding pretest).^4^ Paired samples *t*-tests showed changes in the expected direction, with mostly strong effects for all tasks and subjective measures (Table 1), indicating that participants felt more mentally and physically fatigued, perceived greater effort, and were less motivated. The only exception was motivation in the Footbonaut task, which did not differ significantly before and after the manipulation.

**Table 1:** Descriptive statistics and paired t-tests results for the subjective measures motivation, mental effort, mental fatigue, and physical fatigue in the Stroop test, the LSPT and the Footbonaut task.

	Pre		Post		*t(23)*	*p*	*d*
	*M*	*SD*	*M*	*SD*			
Stroop test							
Motivation	83.00	21.19	73.38	27.33	2.85	0.009	0.58
Mental effort	36.71	23.51	57.83	23.52	−4.55	<0.001	−0.93
Mental fatigue	26.46	18.96	54.46	24.02	−6.20	<0.001	−1.27
Physical fatigue	20.67	16.32	45.38	23.29	−6.65	<0.001	−1.36
LSPT							
Motivation	80.92	24.36	66.50	31.37	3.65	0.001	0.75
Mental effort	34.04	20.80	50.38	20.38	−4.80	<0.001	−0.98
Mental fatigue	31.79	23.75	53.58	21.17	−5.40	<0.001	−1.10
Physical fatigue	35.38	20.12	58.21	21.59	−6.35	<0.001	−1.30
Footbonaut task							
Motivation	89.92	14.13	86.21	22.61	0.78	0.445	0.16
Mental effort	34.50	21.77	54.33	24.83	−3.58	0.002	−0.73
Mental fatigue	32.17	22.87	62.21	25.03	−7.26	<0.001	−1.48
Physical fatigue	36.63	20.26	59.00	4.74	−5.06	<0.001	−1.03

#### 3.2.2 Physiological measures

Since the physical demands of the familiarization task (mentally less demanding) and the mentally demanding Footbonaut task were designed to place comparable physiological demands, no significant differences in HR and BLa between conditions were expected during performance of the Footbonaut task. To test this, separate two-way repeated-measures ANOVAs (Condition × Time of Measurement) were conducted for HR and BLa in each of the three comparisons (familiarization condition vs. Stroop/LSPT/Footbonaut condition). Only interaction effects are reported here (for descriptive and main effects, see ESM 3: Supplementary Tables S4–S6).

In the Stroop comparison, the Condition x Time interaction was not significant for HR, *F*(1.34, 29.57) = 2.34, *p* = 0.130, *η*_p_^2^ = 0.096, or for BLa, *F*(2.23, 46.83) = 1.25, *p* = 0.300, *η*_p_^2^ = 0.056. In the LSPT comparison, the interaction was not significant for HR, *F*(1.34, 29.43) = 1.80, *p* = 0.190, *η*_p_^2^ = 0.076, but reached significance for BLa, *F*(2.19, 39.42) = 6.96, *p* = 0.002, *η*_p_^2^ = 0.279. Similarly, in the Footbonaut comparison, the HR interaction was not significant, *F*(1.86, 40.93) = 0.87, *p* = 0.419, *η*_p_^2^ = 0.038, while for BLa, a significant interaction emerged, *F*(2.57, 54.04) = 4.75, *p* = 0.008, *η*_p_^2^ = 0.184.

Despite the lack of statistical significance in some interactions, the magnitude of the associated effect sizes warranted post-hoc comparisons, which consistently indicated similar physiological trajectories across all conditions.

Before performing the Footbonaut task, HR was comparable between the familiarization and experimental conditions. After the first set of 80 balls, HR increased significantly in all conditions (i.e., indicating higher physical load); however, values were higher in the familiarization condition than in each of the respective experimental conditions. This difference disappeared after the next set in the LSPT and Footbonaut condition. Notably, in the Stroop comparison, HR remained elevated in the familiarization condition up to the fourth time of measurement.

The BLa followed a similar trend: Baseline levels were generally comparable, except for the Stroop comparison, where BLa was already higher in the familiarization condition (i.e., indicating higher physical load). After the first set, BLa increased in all conditions, with significantly higher values in the familiarization condition than in the respective experimental conditions. These differences disappeared by the third time of measurement, except again in the Stroop comparison, where elevated BLa levels in the familiarization condition also persisted at the fourth time of measurement.

In sum, some interaction effects emerged. These were contrary to expectations and primarily driven by unexpectedly higher HR and BLa responses in the familiarization condition, particularly after the first set of balls. However, taken together, the findings do not suggest a substantial overall difference in physical load between the conditions.

### 3.3 Main analyses

It was hypothesized that the experimental manipulation in the Footbonaut task would impair accuracy and response/processing times in (1) cognitive performance (Stroop task: more errors, longer response times), (2) soccer-specific performance (LSPT: longer penalty times, longer processing times), and (3) task-specific performance (Footbonaut: lower accuracy, longer response times). Further, we assumed that the experimental manipulation in the Footbonaut would differentially affect performance in the subsequent tasks (Stroop, LSPT, and Footbonaut).

To test these hypotheses, the different performance parameters were first z-standardized. The z-standardization was based on the mean and the pooled standard deviation computed from the aggregated pre- and posttest scores. Since for the Footbonaut task passing performance, higher values in the posttest compared to the pretest represent a performance improvement, whereas for the number of errors in the Stroop task and the penalty time in the LSPT, higher values represent a deterioration in accuracy performance, the signs of the z-values were reversed for passing performance in order to make this value comparable to the other two.

Two separate two-way repeated measures ANOVAs [Condition (Stroop vs. LSPT vs. Footbonaut) × Time (pre vs. post)] were then computed, one for accuracy and one for response time parameters. For all hypotheses, interaction results were examined with *post hoc* tests (comparison pre-post Stroop, pre-post LSPT, pre-post Footbonaut). Descriptive statistics and further details on the main effects can be found in ESM 3: Supplementary Tables S7, S8.

#### 3.3.1 Accuracy

**Figure 5:**
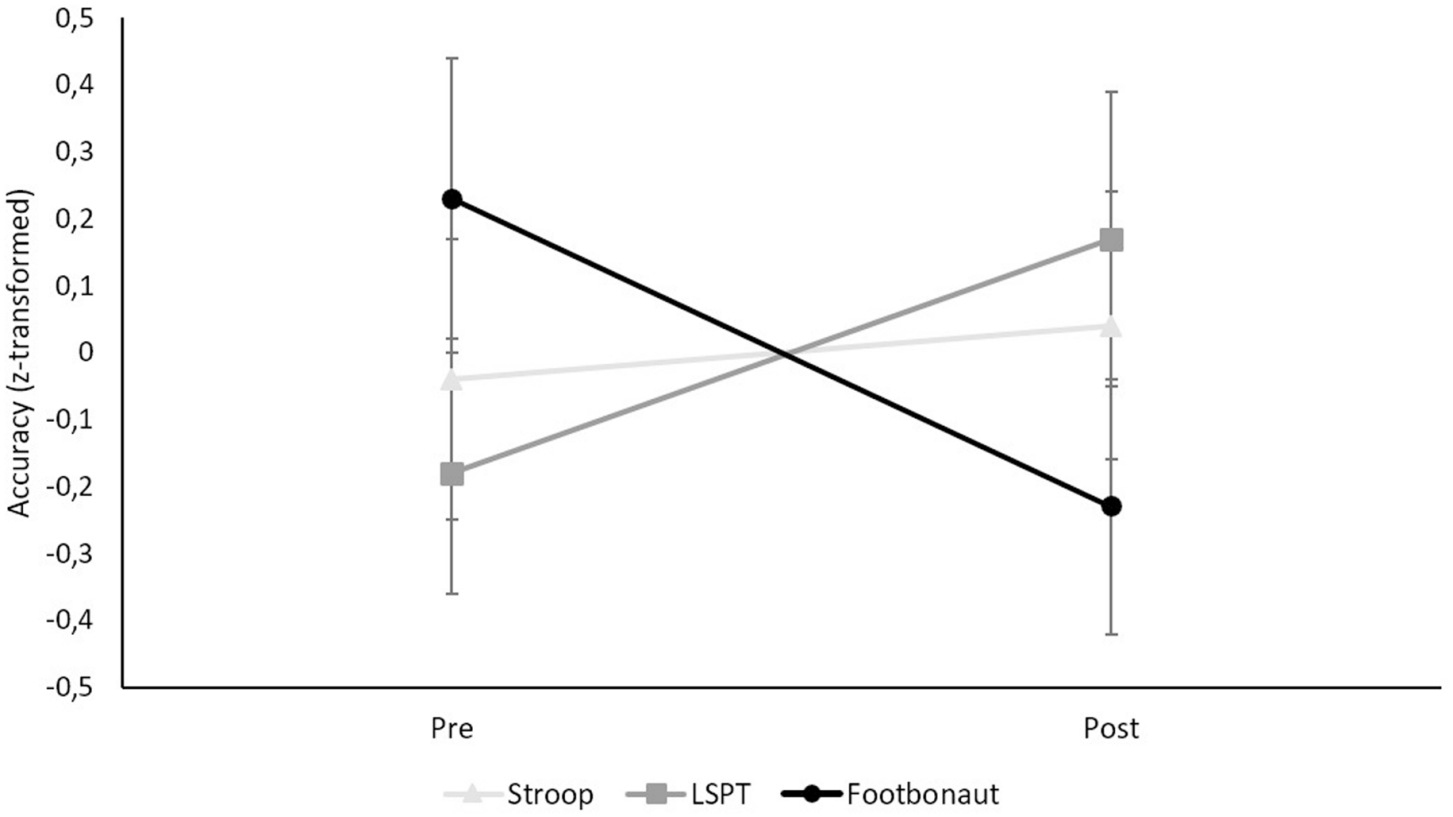
Condition-specific differences in the accuracy (z-transformed).

The repeated-measures ANOVA [Condition (Stroop vs. LSPT vs. Footbonaut) × Time (pre vs. post)] for the z-standardized accuracy parameters (Stroop: errors, LSPT: penalty time, Footbonaut: passing performance) revealed a significant interaction effect [*F*(2, 46) = 4.70, *p* = 0.014, *η*_p_^2^ = 0.170; Figure 5]. Inspection of the post-hoc tests indicated that the number of errors committed in the Stroop task did not change significantly from pre- to posttest (*p* = 0.998). The same was true for penalty times in the LSPT (*p* = 0.388), and passing performance in the Footbonaut task (*p* = 0.135). Although none of the post-hoc tests revealed statistically significant changes, the significant interaction effect is likely due to the fact that participants showed improved passing performance in the Footbonaut after the manipulation (i.e., more accurate passes), but had higher penalty times in the LSPT (i.e., increased penalty times), while the number of errors in the Stroop task remained largely unchanged (Figure 5).

#### 3.3.2 Response time

**Figure 6:**
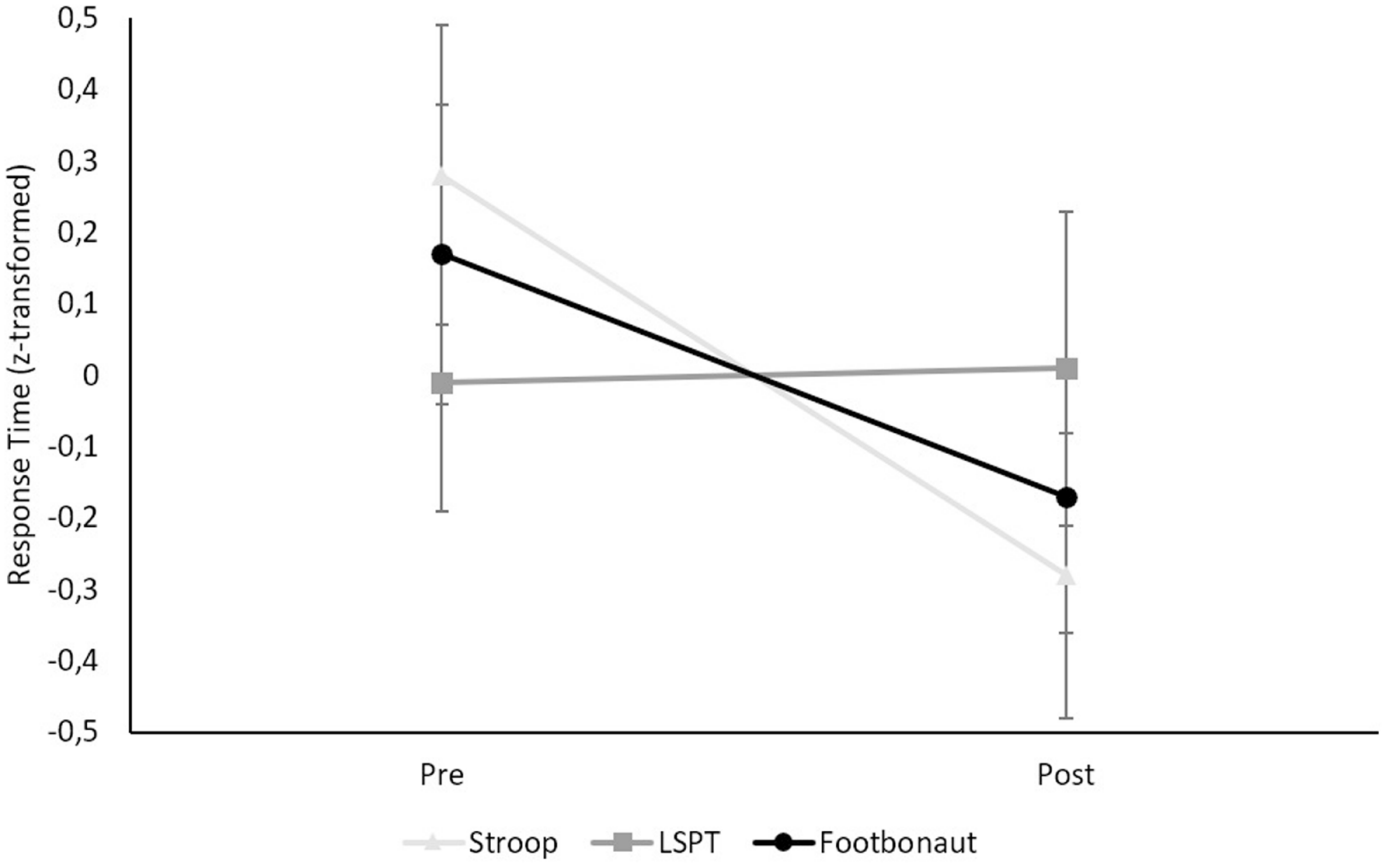
Condition-specific differences in the response time (z-transformed).

The repeated-measures ANOVA [Condition (Stroop vs. LSPT vs. Footbonaut) × Time (pre vs. post)] for the z-standardized response time parameters (Stroop: response latencies, LSPT: movement time, Footbonaut: processing speed) revealed no significant interaction effect, *F*(2, 46) = 2.90, *p* = 0.065, η_p_^2^ = 0.170 (Figure 6), although the effect size was large. Inspection of the post-hoc tests revealed that response latencies in the Stroop task were significantly faster after the manipulation than before (i.e., decreased response latencies, *p* = 0.026). For movement time in the LSPT (*p* > 0.999) and processing speed in the Footbonaut (*p* = 0.398), no significant changes were found, suggesting that the manipulation task did indeed have differential effects on the response time across the three tasks.

## 4 Discussion

Over the years, researchers have employed a variety of (cognitive) tasks to induce MF, with the 30-min (modified) Stroop task emerging as one the most widely used and established methods (Van Cutsem and Marcora, 2021; Sun et al., 2022). However, the applicability of such cognitive tasks to real-world settings has been questioned, as their lack of sport-specificity and low ecological validity limit their transferability to the mental demands athletes face during training and competition (Bian et al., 2022; Habay et al., 2021). To overcome this methodological limitation in MF research, researchers have recently developed more sport-specific manipulation tasks (e.g., modified Footbonaut task; Weiler et al., 2025b). The central aim of the present study was to further validate the mentally demanding version of the Footbonaut task developed by Weiler et al. (2025b) to induce soccer-specific MF. The study examined the effects of this Footbonaut task on performance across three different task types: the Footbonaut (identical task; task-specific), the LSPT (soccer-specific; near-transfer) and the Stroop task (general cognitive; far-transfer). These performance tests were administered both before and after the manipulation of MF via the Footbonaut. Accordingly, declines in performance from pre- to posttest were expected across all three tasks. Furthermore, given previous inconsistencies in far-transfer effects of soccer-specific manipulation tasks on general cognitive performance (e.g., Ehmann et al., 2022; Gobet and Sala, 2023), the present study examined whether the three task types were affected to different degrees by the Footbonaut manipulation.

Although a significant interaction effect was observed for the accuracy parameters, for the Stroop task, contrary to our expectations, the number of errors did not significantly change from pre- to posttest. Likewise, no significant effect was found on the accuracy in the LSPT, although descriptively, performance declines in penalty times were observed following the manipulation task. An opposite pattern, indicating improved average passing performance, was observed for accuracy in the Footbonaut. However, this effect also did not reach statistical significance. Conversely to the accuracy parameters, there was no significant interaction effect for the response time parameters. Interestingly, post-hoc tests nevertheless revealed a significant decrease in average response time regarding the Stroop task following the manipulation, indicating that participants responded faster to the stimuli after the MF induction than before. By contrast, the processing time for the LSPT and the average processing speed in the Footbonaut remained unaffected. Taken together, it cannot be concluded that the different task types were negatively affected by the manipulation task in terms of accuracy or response time, nor that they were affected to different degrees.

The fact that no negative effects of the Footbonaut were observed across performance in the three different task types raises the question of whether the Footbonaut task is a suitable MF task. In previous research, cognitive tasks such as the Stroop task have been successfully employed to investigate the effects of MF on sports performance (e.g., Smith et al., 2016; Trecroci et al., 2020). These tasks are typically characterized by their repetitive and monotonous nature, as well as a low degree of contextual relevance (Radtke et al., 2023; Thompson et al., 2019; Thompson C. et al., 2020). Therefore, it is unclear whether impaired performance after having worked on a monotonous cognitive task is caused by the mental effort required to solve the respective cognitive task or whether it is–at least partially–caused by boredom and reduced intrinsic motivation to engage in the (following) task (Martarelli et al., 2023; Thompson C. et al., 2020). In the current study, as opposed to other studies which have administered the Stroop task (e.g., Thompson C. et al., 2020), players reported high levels of motivation and rather low levels of boredom regarding the Footbonaut task developed to induce MF. It is reasonable to assume that the high contextual interference of the Footbonaut task might have increased participants’ engagement and simultaneously minimized confounding effects of boredom or reduced motivation, which may have decisively caused performance impairments in previous studies (Milyavskaya et al., 2021; Thompson C. et al., 2020). Consequently, it can be assumed that the Footbonaut manipulation task was not mentally demanding enough to induce MF in the soccer players. Due to the high levels of motivation and engagement typically associated with sport-specific manipulation tasks (Thompson C. et al., 2020), future studies may need to increase the mental complexity and durations of such tasks in order to enhance the mental demands and, in turn, effectively induce MF. The complexity of the Footbonaut task (Habay et al., 2021; O’Keeffe et al., 2020) could be further increased by incorporating dual-task paradigms, in which athletes simultaneously perform a motor and a cognitive task, or by integrating auditory distractors in addition to visual cues (Habay et al., 2021; O’Keeffe et al., 2020). Both approaches may increase the variability of instructions and thereby enhance the mental load during the task. Additionally, the task instructions could be varied at shorter intervals to prevent automated execution and to maintain cognitive engagement and mental load throughout the task (Weiler et al., 2025b). Furthermore, future studies may consider implementing adaptive difficulty levels (e.g., maintaining a success rate between 40–60%) that dynamically adjust according to the athletes’ performance (Li et al., 2025). This approach can help maintain a continuously challenging but achievable mental load, thereby sustaining motivation and engagement throughout the task. Another possibility to increase the mental demands of the task could be achieved by extending its duration. It may be necessary to extend the task duration beyond 30 min, particularly for high-skilled athletes who are used to sustaining mental demands over extended periods of time, such as during 90-min soccer matches (Boat et al., 2020; Ciocca et al., 2022). Beyond that, future research should incorporate gaze behavior measures to gain a more comprehensive understanding of participants’ visual engagement during mentally fatiguing tasks. Specifically, assessments of pupil diameter and pupil dynamics may provide deeper insights into task engagement and disengagement, as well as allow for a more accurate identification of the onset of MF (Hopstaken et al., 2015a, 2015b).

In addition, there are further aspects that may have had a diminishing effect on MF, which can be attributed to methodological limitations of the current study. For instance, given that regular breaks were implemented during the execution of the Footbonaut task to assess physiological parameters, it cannot be ruled out that these breaks allowed the players to partially recover from the mentally effortful task (Thompson C. et al., 2020). As a result, the intended accumulation of MF may have been attenuated. Moreover, even though the instructions between the different sets being performed in the Footbonaut changed, players may have become accustomed to the passing task over time. They may have developed strategies while performing the passes, such as adapting their visual search strategies or employing a more efficient and economic passing technique (passing vs. shooting). This would have reduced the mental demands of the task (Fortes et al., 2022). To address this potential limitation, future studies should restrict the time available for performing each pass to ensure sustained mental effort. For example, light signals could be extinguished after a predefined period, requiring soccer players to process the stimuli within a limited time frame to ensure the trial is deemed valid.

An additional aspect that warrants critical consideration concerns the inconsistencies in control measures applied in the current study. The subjective measures aligned with our initial expectations. Following the execution of the mentally effortful manipulation task in the Footbonaut, players reported lower levels of motivation, alongside increased levels of mental effort, mental fatigue, and physical fatigue during the posttest tasks compared to the pretest tasks. However, these self-reported changes were not reflected by corresponding declines in objective performance outcomes and can accordingly only be interpreted as preliminary indicators of the occurrence of MF (for a discussion, see also Englert, 2025). One potential explanation for these unexpected findings is that the current sample might have differed from previous samples in terms of specific personality traits (e.g., MF-susceptibility; see also Skau et al., 2021) or in terms of their physical fitness (e.g., André et al., 2025). In a recent study by Daneshgar-Pironneau et al. (2025), it was observed that the physical performance of athletes was not affected by a preceding Stroop task. In contrast, non-athletes demonstrated a significant decline in performance during a state of MF. In view of the fact that the subjects of the present study were classified as 2 tier athletes (McKay et al., 2022), it is recommended that future research should consider the possibility of expertise as a potential alternative explanation (Lu et al., 2025; Wang et al., 2024).

Another issue concerns the physiological parameters, which aimed to detect unintended effects of physical effort. BLa and HR were measured between the different sets of the manipulation task in the Footbonaut (T2–T4), as well as during familiarization (T1) while performing the standard Footbonaut task. Although the tasks were matched in terms of duration and physical demands, contrary to expectations, higher BLa and HR responses were observed during the familiarization phase, particularly after the first set of ball passes in the Footbonaut. Therefore, the observed differences in physiological responses between the manipulation and standard tasks might not be driven primarily by task type, but rather by the order in which the tasks were administered. Specifically, the standard task was always completed during the familiarization phase at the first time of measurement, whereas the manipulation task was conducted only at subsequent times of measurement, when the players had already become familiar with the general demands of the Footbonaut task. The fact that the largest differences in the physiological markers occurred following the first set of ball passes further supports the assumption of a possible order effect. This is additionally supported by the findings of Weiler et al. (2025b), who also compared physiological demands using BLa and HR between the standard task and a modified manipulation task. In their study, the conditions were conducted in a randomized order at the second and third time of measurement. No significant differences in BLa or HR were found between the two task conditions, indicating, as expected, comparable physiological demands. Nevertheless, based on these results, it cannot be ruled out that unintended physical fatigue may have confounded the observed outcomes. Future studies could therefore consider adopting EEG, as it may serve as a sensitive biomarker for detecting mental fatigue (Van Cutsem and Marcora, 2021; Goodman et al., 2025). Due to its high temporal resolution, EEG could meaningfully complement existing physiological measures by capturing both the onset and the dynamics of MF more precisely.

Although the results of the present study were not conclusive, the study design itself provides valuable implications for both research and applied contexts in sport. By systematically assessing the effects of a mentally fatiguing, sport-specific manipulation task across task-specific, near-transfer, and far-transfer levels, the study aims to explore whether MF induced by soccer-specific demands impacts performance solely in sport-related contexts (task-specific, near-transfer effects) or also extends to general cognitive domains (far-transfer effects). This provides a more differentiated understanding of how MF may affect performance differently across distinct domains. For instance, if MF originating from playing football (e.g., via the Footbonaut task) primarily affects performance in identical or similar sport-specific tasks, but not in unrelated, general cognitive tasks, this could suggest that acute sport-related MF has limited impact on non-sport activities, such as academic or occupational functioning. Conversely, if such fatigue also impairs performance in general cognitive tasks, this may indicate that sport-induced MF could carry over to negatively affect off-field performance. Vice versa, this line of research also opens up the question of whether MF stemming from daily life (e.g., academic pressure or occupational responsibilities) might impair sport-specific performance. This could be especially relevant for athletes at the semi-professional level who balance dual careers. Understanding these dynamics may help coaches, sport psychologists, and practitioners make more informed decisions regarding mental recovery, mental load management, and the scheduling of mentally demanding tasks before competitions, including consideration of everyday mental demands such as academic or occupational requirements.

## 5 Conclusion

Taken together, the present study did not provide conclusive evidence that the modified manipulation task in the Footbonaut induced MF in the participating soccer players. Therefore, performance across the three task types (Footbonaut, LSPT, and Stroop task) was neither significantly impaired nor affected to varying degrees following the manipulation. Nevertheless, several important insights and methodological advancements have emerged from this study. In particular, the usage of a sport-specific manipulation task like the Footbonaut represents a promising step toward improving the ecological validity of MF research in sports: Compared to traditional MF protocols, the Footbonaut task was associated with high levels of motivation and low levels of boredom among players, suggesting that it successfully maintained engagement while reducing confounding effects such as boredom or demotivation. Moreover, although no significant performance declines were observed, subjective measures indicated preliminary signs of MF, highlighting the importance of a multimodal assessment approach. Additionally, the study offers important directions for refining future research, including increasing the mental demands of the task through greater complexity or extended duration, limiting recovery periods during task execution, and incorporating gaze-based metrics to more precisely track the onset of MF. Overall, the study highlights the importance of methodological innovations in advancing the understanding of MF in sports and offers concrete recommendations designing more conclusive and rigorous future experimental protocols.

## Data Availability

The raw data supporting the conclusions of this article will be made available by the authors, without undue reservation.

## Footnotes

^1^ https://aspredicted.org/px65-ryxv.pdf

^2^ https://www.randomization.com

^3^We did not preregister the analysis of possible order effects. However, we believe that this analysis provides valuable additional information.

^4^Due to an error in the preregistration, it was specified that boredom would also be analyzed here. However, boredom was only measured in relation to the MF induction task, not in relation to the tasks used to operationalize the dependent variables. Therefore, this variable could not be analyzed.

## Supplementary material

Supplementary Data Sheet 1 Instructions for the Footbonaut and Rules of the LSPT.

Supplementary Data Sheet 2 Results regarding order effects.

Supplementary Data Sheet 3 Additional information on the results.
