# Supplementary material for: Investigating the isolated effects of a soccer-specific mental fatigue manipulation on different task types
Source: Front Psychol. 2025 Sep 18;16:1655221. doi: 10.3389/fpsyg.2025.1655221 (PMC12489948; doi:10.3389/fpsyg.2025.1655221)
Supplement: Supplementary Data Sheet 1 — Instructions for the Footbonaut and Rules of the LSPT. [file Data_Sheet_1.pdf]

Electronic Supplemental Material 1 (ESM 1)

Instructions – Instructions of the experimental condition for the Footbonaut

1st series: "Your task in the first series is to play the blue light."

2nd series: "Your task in the second series is to play the green light that is closer to the blue light."

Series 3: "Your task in the third series is to play the green light that is further away from the blue light."

Series 4: "Your task in the fourth series is to play the green light that is closer to the blue light."

LSPT Scoring – Detailed rules for the bonus and penalty times of the LSPT

Incorrect passes were penalized as follows: 1) missing the bench completely or playing the incorrect bench (+ 5 seconds), 2) missing the color target area (+ 3 seconds), 3) handling the ball with the hand, arm or shoulder area (+ 3 seconds), 4) passing the ball outside the passing zone (+ 2 seconds), 5) touching the cones with the ball (+ 2 seconds), and 6) requiring more than 43 seconds to complete the test. The players were also able to improve their total passing time by hitting the white stripe within the colored target area (- 1 second).
