# Supplementary material for: Investigating the isolated effects of a soccer-specific mental fatigue manipulation on different task types
Source: Front Psychol. 2025 Sep 18;16:1655221. doi: 10.3389/fpsyg.2025.1655221 (PMC12489948; doi:10.3389/fpsyg.2025.1655221)
Supplement: Supplementary Data Sheet 2 — Results regarding order effects. [file Data_Sheet_2.pdf]

ESM 2

Results for all analyses regarding order effects between T2, T3 and T4

Table S1

Estimated marginal means and results of the two-way repeated measures ANOVA (Testing Session (T2 vs. T3 vs. T4) x Subjective measure (Mental effort vs. Mental fatigue vs. Physical fatigue vs. Boredom)) for the subjective measures regarding the manipulation task in the Footbonaut

Variable	T2		T3		T4		Total (main effect S)		ANOVA				
	M	*SE*	*M*	*SE*	*M*	*SE*	*M*	*SE*	Effect	*df*	F	*p*	η^2_p
Mental effort	57.96	4.02	57.38	3.67	53.33	4.46	56.22	3.34	TS	2, 46	0.27	.768	0.011
Mental fatigue	54.88	4.80	51.68	4.55	52.75	4.96	53.10	3.92	S	3, 69	33.62	< .001	0.594
Physical fatigue	59.63	4.75	56.00	4.14	54.50	4.47	56.71	3.74	TS x S	6, 138	0.68	.666	0.029
Boredom	21.13	5.37	21.68	5.12	25.96	5.72	22.92	4.39					
Total (main effect TS)	48.40	3.50	46.68	3.14	46.64	3.53							

Note. *N* = 24; TS = Testing Session (T2 vs. T3), S = Subjective measure (Mental effort vs. Mental fatigue vs. Physical fatigue vs. Boredom).

Table S2

Estimated marginal means and results of the two-way repeated measures ANOVA (Testing Session (T2 vs. T3 vs. T4) x Time of Assessment (Pre vs. Post)) for the accuracy-related parameters

Variable	Pre		Post		Total (main effect T)		ANOVA				
	M	*SE*	*M*	*SE*	*M*	*SE*	Effect	*df*	*F*	*p*	η^2_p
T1	0.04	0.19	0.19	0.21	0.12	0.16	TS	2, 46	0.49	.615	0.021
T2	0.10	0.25	0.03	0.24	0.06	0.22	T	1, 23	0.17	.689	0.007
T3	−0.13	0.17	−0.08	0.18	−0.11	0.15	TS x T	2, 46	0.24	.788	0.010
Total (main effect)	0.01	0.13	0.05	0.14							

Note. *N* = 24; TS = Testing Session (T2 vs. T3 vs. T4), T = Time of Assessment (Pre vs. Post).

Table S3

Estimated marginal means and results of the two-way repeated measures ANOVA (Testing Session (T2 vs. T3 vs. T4) x Time of Assessment (Pre vs. Post)) for the response time-related parameters

Variable	Pre		Post		Total (main effect T)		ANOVA				
	M	*SE*	*M*	*SE*	*M*	*SE*	Effect	*df*	*F*	*p*	η^2_p
T1	0.18	0.17	−0.07	0.14	0.05	0.14	TS	2, 46	0.29	.752	0.012
T2	0.06	0.25	−0.14	0.22	−0.04	0.22	T	1, 23	4.81	.039	0.173
T3	0.01	0.22	−0.23	0.17	−0.11	0.16	TS x T	2, 46	0.02	.983	< .001
Total (main effect)	0.08	0.14	−0.15	0.13							

Note. *N* = 24; TS = Testing Session (T2 vs. T3 vs. T4, T = Time of Assessment (Pre vs. Post).
