# Supplementary material for: Investigating the isolated effects of a soccer-specific mental fatigue manipulation on different task types
Source: Front Psychol. 2025 Sep 18;16:1655221. doi: 10.3389/fpsyg.2025.1655221 (PMC12489948; doi:10.3389/fpsyg.2025.1655221)
Supplement: Supplementary Data Sheet 3 — Additional information on the results. [file Data_Sheet_3.pdf]

ESM 3

Additional information on the results

Table S4

Estimated marginal means and results of the two-way repeated measures ANOVA (Condition (Control vs. Stroop) x Assessments in the Footbonaut (Baseline vs. 1 vs. 2 vs. 3 vs. 4)) for the physiological measures of the Footbonaut

Variable	Control Condition		Stroop Condition		Total (main effect T)		ANOVA				
	M	*SE*	*M*	*SE*	*M*	*SE*	Effect	*df*	*F*	*p*	η^2_p
HR											
Baseline	96.17	2.79	91.96	5.72	94.07	3.80	C	1, 22	20.33	< .001	.480
1	173.78	2.73	159.35	3.21	166.57	2.70	T	4, 88	408.78	< .001	.949
2	170.09	3.00	159.87	3.16	164.98	2.76	C x T	4, 88	2.34	.061	.096
3	171.22	2.99	159.96	3.02	165.59	2.81					
4	169.44	2.90	161.17	2.94	165.31	2.66					
Total (main effect C)	156.14	2.63	146.46	3.02							
BLa											
Baseline	2.33	0.33	1.08	0.12	1.71	0.18	C	65.53, 4.07	16.10	< .001	0.434
1	4.12	0.64	2.73	0.56	3.43	0.55	T	20.55, 1.62	12.69	< .001	0.377
2	3.36	0.58	2.15	0.49	2.76	0.51	C x T	0.89, 0.72	1.25	0.30	0.056
3	2.75	0.48	1.82	0.35	2.29	0.39					
4	2.30	0.37	1.62	0.29	1.96	0.32					
Total (main effect C)	2.97	0.45	1.88	0.34							

Note. *N* = 24; C = Condition (Control vs. Stroop), T = Time of Assessment in the Footbonaut (Baseline vs. 1 vs. 2 vs. 3 vs. 4).

Table S5

Estimated marginal means and results of the two-way repeated measures ANOVA (Condition (Control vs. LSPT) x Assessments in the Footbonaut (Baseline vs. 1 vs. 2 vs. 3 vs. 4)) for the physiological measures of the Footbonaut

Variable	Control Condition		LSPT Condition		Total (main effect T)		ANOVA				
	M	*SE*	*M*	*SE*	*M*	*SE*	Effect	*df*	*F*	*p*	η^2_p
HR											
Baseline	96.17	2.79	93.70	3.64	94.94	2.34	C	1, 22	13.37	.001	0.378
1	173.78	2.73	162.09	3.27	167.94	2.63	T	4, 88	< .001	< .001	0.966
2	170.09	3.00	163.04	3.00	166.57	2.71	C x T	4, 88	1.80	.136	0.076
3	171.22	2.99	161.22	3.31	166.22	2.87					
4	169.44	2.90	161.35	3.32	165.39	2.78					
Total (main effect C)	156.14	2.63	148.28	2.65							
BLa											
Baseline	2.12	0.34	2.16	0.34	2.19	0.32	C	1, 18	6.95	.017	0.279
1	3.91	0.68	2.37	0.42	3.14	0.48	T	4, 72	11.94	< .001	0.399
2	3.22	0.65	1.82	0.37	2.52	0.47	C x T	4, 72	6.96	< .001	0.279
3	2.61	0.51	1.49	0.26	2.05	0.35					
4	2.16	0.40	1.43	0.25	1.79	0.30					
Total (main effect C)	2.82	0.49	1.85	0.31							

Note. *N* = 24; C = Condition (Control vs. LSPT), T = Time of Assessment in the Footbonaut (Baseline vs. 1 vs. 2 vs. 3 vs. 4).

Table S6

Estimated marginal means and results of the two-way repeated measures ANOVA (Condition (Control vs. Footbonaut) x Assessments in the Footbonaut (Baseline vs. 1 vs. 2 vs. 3 vs. 4)) for the physiological measures of the Footbonaut

Variable	Control Condition		Footbonaut Condition		Total (main effect T)		ANOVA				
	M	*SE*	*M*	*SE*	*M*	*SE*	Effect	*df*	*F*	*p*	η^2_p
HR											
Baseline	96.17	2.79	90.09	4.68	93.13	3.18	C	1, 22	16.54	< .001	0.429
1	173.78	2.73	163.22	3.22	168.50	2.73	T	4, 88	492.31	< .001	0.957
2	170.09	3.00	163.39	2.97	166.74	2.80	C x T	4, 88	0.76	.554	0.033
3	171.22	2.99	161.78	3.22	166.50	2.83					
4	169.44	2.90	163.04	3.05	166.24	2.78					
Total (main effect C)	156.14	2.63	148.30	2.80							
BLa											
Baseline	2.20	0.29	2.08	0.29	2.14	0.23	C	1, 21	8.14	.010	0.279
1	4.10	0.64	2.62	0.44	3.36	0.48	T	4, 84	13.01	< .001	0.383
2	3.42	0.58	2.13	0.38	2.77	0.45	C x T	4, 84	5.37	< .001	0.204
3	2.81	0.49	1.76	0.25	2.29	0.34					
4	2.34	0.38	1.41	0.14	1.87	0.23					
Total (main effect C)	2.98	0.45	2.00	0.27							

Note. *N* = 24; C = Condition (Control vs. Footbonaut), T = Time of Assessment in the Footbonaut (Baseline vs. 1 vs. 2 vs. 3 vs. 4).

Table S7

Estimated marginal means and results of the two-way repeated measures ANOVA (Condition (Stroop vs. LSPT vs. Footbonaut) x Time of Assessment (Pre vs. Post)) for the accuracy-related parameters

Variable	Pre		Post		Total (main effect T)		ANOVA				
	M	*SE*	*M*	*SE*	*M*	*SE*	Effect	*df*	*F*	*p*	η^2_p
Stroop	−0.04	0.21	0.04	0.20	< .001	0.19	C	2, 46	< .001	> .999	< .001
LSPT	−0.18	0.18	0.17	0.22	< .001	0.19	T	1, 23	0.01	.921	< .001
Footbonaut	0.23	0.21	−0.23	0.19	< .001	0.16	C x T	2, 46	4.70	.014	.170
Total (main effect)	0.00	0.13	−0.00	0.14							

Note. *N* = 24; C = Condition (Stroop vs. LSPT vs. Footbonaut), T = Time of Assessment (Pre vs. Post).

Table S8

Estimated marginal means and results of the two-way repeated measures ANOVA (Condition (Stroop vs. LSPT vs. Footbonaut) x Time of Assessment (Pre vs. Post)) for the response time-related parameters

Variable	Pre		Post		Total (main effect T)		ANOVA				
	M	*SE*	*M*	*SE*	*M*	*SE*	Effect	*df*	*F*	*p*	η^2_p
Stroop	0.28	0.22	−0.28	0.17	< .001	0.19	C	2, 46	< .001	> .999	< .001
LSPT	−0.01	0.22	0.01	0.19	< .001	0.17	T	1, 23	7.52	.012	0.246
Footbonaut	0.17	0.22	−0.17	0.18	< .001	0.18	C x T	2, 46	2.90	.065	0.112
Total (main effect)	0.15	0.15	−0.15	0.13							

Note. *N* = 24; C = Condition (Stroop vs. LSPT vs. Footbonaut), T = Time of Assessment (Pre vs. Post).
